# Social Media and Multiple Sclerosis in the Posttruth Age

**DOI:** 10.2196/ijmr.7879

**Published:** 2017-09-27

**Authors:** Luigi Lavorgna, Roberta Lanzillo, Vincenzo Brescia Morra, Gianmarco Abbadessa, Gioacchino Tedeschi, Simona Bonavita

**Affiliations:** ^1^ 1st Clinic of Neurology University of Campania Naples Italy; ^2^ Clinic of Neurology Department of Neurosciences, Reproductive Sciences and Odontostomatology Federico II University Naples Italy

**Keywords:** social media, multiple sclerosis, social network, digital health, eHealth, Web medicine

## Abstract

Over the last few decades, patients have increasingly been searching for health information on the Internet. This aspect of information seeking is really important, especially for people affected by chronic pathologies and require lifelong treatment and management. These people are usually very well informed about the disease, but are nonetheless vulnerable to hopes of being cured or saved, often amplified by misinformation, myths, legends, and therapies that are not always scientifically proven. Many studies suggest that some individuals prefer to rely on the Internet as their main source of information, often hindering the patient-doctor relationship. This is why a professional approach is imperative in this posttruth age, in order to maintain confidentiality, honesty, and trust in the medical profession.

## Background

Many patients with multiple sclerosis (MS) have expressed a need for more information and support [1,2]. The Internet is now an important source of health and medical information and its accessibility often makes it the first step in obtaining information about diseases and their treatment. Patients with MS, their families, and/or caregivers use the Web in everyday life as a source of medical information and to interact with others who are living with the same condition [3-5]. The Web can also be a source of comfort and support through the exchange of experiences, opinions, and emotions [6]. Patients and caregivers can find a wide range of opportunities for peer interactions and learning online. However, we need to differentiate between the various levels of validity and objectivity of the information that they might find online.

A group of neurologists, psychologists, anthropologists, science journalists, and legal authorities met in Naples, Italy, in November 2016 during the *Social Media and Multiple Sclerosis (SMMS): Communities of Practice* meeting. They discussed Internet usage by MS patients seeking health- and disease-related information for self-care and self-management purposes in the “posttruth” age. In this paper, we stress the need to provide patients and their caregivers with tools for prudent and productive Web navigating [7] and ensure that they can find and share valid information through their social media interactions.

Entering a few keywords into a search engine returns a huge number of hits; however, it is critical that patients and caregivers be able to recognize information that is potentially incorrect or only partially correct. The ranking that a search engine assigns to search results is determined by algorithms inherent to the program and may be based on the number of site interactions or the number of times that a site is linked or referenced. Thus, the choice of a Web search engine also influences the retrieved results [8].

Internet users should know that there are people or groups interested in spreading information that is not based on scientific evidence. Users should also be able to recognize links to sponsored content. Moreover, they should understand that queries made through a search engine are not as “independent” as they might think: results are influenced by stored information about previous searches performed on the computer. Authoritative information sources are preferred, such as scientific foundations and national and international scientific societies.

An early assessment of the quality of online medical information conducted in 1997 on pediatric fever revealed that only one in 10 websites provided complete and completely accurate information [9]. Subsequently, criteria were proposed to standardize the assessment of websites. Two types of criteria were considered: direct and surrogate. Direct criteria, the gold standard for assessing quality, include determining the accuracy and completeness of the information by experts. However, this type of verification is difficult to obtain because it involves expert intervention. Surrogate criteria are characteristics that tend to be associated with high-quality information. These include identifying the website owners and sponsors, providing dates for when the information was updated, supporting the information with bibliographic references to literature published in peer-reviewed medical journals, identifying the authors as medical professionals and indicating their affiliations, disclosing any conflicts of interest, and providing links to the websites of disease-related medical or scientific associations [10]; for example, websites could apply the publishing benchmarks from the Journal of the American Medical Association, which consider authorship, date of most recent update, references, and disclosure of conflicts of interest [11].

Meanwhile, several organizations provide website logos, badges, or “seals of approval” to be displayed only on websites that meet specific criteria [12]. The first organization to provide such a service was the nonprofit Health On the Net (HON) Foundation, established in 1995 to promote reliable online health information and to protect citizens from misleading health information [13]. The HON criteria are summarized in their code of conduct (see Textbox 1). Their website provides information and services that are tailored to patients, caregivers, medical professionals, or website developers.

In addition, the HON website provides an Internet search portal—HON Search—through which it is possible to focus search results on medical information that meets quality criteria. This can significantly improve search effectiveness by excluding advertisements and nonrelated websites from the search results. Results can be further filtered for interest to patients and caregivers or to medical professionals. Such guidance may help information seekers avoid some of the pitfalls of searches.

Another important initiative in this area is the DISCERN Project, which is based at the University of Oxford, Division of Public Health and Primary Health Care, Institute of Health Sciences [14]. Project members developed the DISCERN tool [15], which comprises guidelines for analyzing information on treatment choices that are applicable to a wide range of topics by users at many levels (see Textbox 2). The tool provides an index of the quality of medical information that is useful for patients and caregivers, as well as providing guidance to website content developers. The DISCERN website contains a reference guide that should only be used once one is acquainted with the full DISCERN instrument.

We have summarized the minimal requirements of a medical/scientific website into the following schema (see Textbox 3) and suggest that this information should be provided to MS patients and their caregivers.

We also suggest that the patient or caregiver be informed about the existence of HON and the DISCERN initiatives and be encouraged to use these services.

**Summary of the eight components of the Health On the Net Code of Conduct: criteria for obtaining website certification.**

1. Authority: indicate the qualification of the authors.

2. Complementarity: information should support, not replace, the doctor-patient relationship.

3. Confidentiality: respect the privacy and confidentiality of personal data submitted to the site by the visitor.

4. Attribution: cite the source(s) of published information, date, and medical and health pages.

5. Justification: site must back up claims relating to benefits and performance.

6. Professionalism: accessible presentation and accurate email contact.

7. Transparency of financing: identify funding resources.

8. Advertising: clearly distinguish advertising from editorial content.

**DISCERN Project: summary of characteristics associated with good-quality information about treatment choices.**

A good-quality publication about treatment choices will do the following:

- Have explicit aims
- Achieve its aims
- Be relevant to consumers
- Make sources of information explicit
- Make date of information explicit
- Be balanced and unbiased
- List additional sources of information
- Refer to areas of uncertainty
- Describe how treatment works
- Describe the benefits of treatment
- Describe the risks of treatment
- Describe what would happen without treatment
- Describe the effects of treatment choices on overall quality of life
- Make it clear there may be more than one possible treatment choice
- Provide support for shared decision-making

**Minimal requirements of a medical/scientific website.**

A medical/scientific website must do the following:

- Use the correct vocabulary, be clear, and be easily understandable
- Clearly identify its managers and sponsors
- Have an editorial board responsible for scientific content
- Provide bibliographic support for all news published so that personal opinion—even from an expert—is clearly discernible from peer-reviewed scientific literature
- Clearly distinguish sponsored content
- Indicate when its content was last updated so the visitor knows if it is outdated
- Clearly state their privacy policy

## Social Media as a Support Tool for People With Multiple Sclerosis and Their Caregivers

An online community or support group allows exchange of experiences and information among group members that may be helpful to patients and caregivers [16-18]. Online communities are characterized by communication that can be synchronous (eg, instant message, chat, and video chat) or asynchronous (eg, forums and blogs). Both forms of communication can be used to give and receive support and to interact with people who are sharing the same life experiences. Social media can also serve as a research tool to collect anonymous information for studying many aspects of the MS patient’s journey [19,20].

We have collected some of the features found in online communities that can be instrumental for meeting the need for support and interaction that a person living with MS may be experiencing:

1. Public profiles. Communities with open profiles that are visible to everyone help users to find each other easily and to share personal information and experiences related to MS.
2. Messages. Communities that allow exchange of different types of messages meet the diverse needs of people with MS:
  - Connections among individual users (ie, chat)
  - Messages among established contacts (ie, contact lists, friend requests, and private messages)
  - Peer counseling (ie, people with MS who volunteer their time to help others with MS)
3. Forum. These are forms of asynchronous interaction and communication that must be moderated to prevent abuses; moderation also serves to avoid the spread of incorrect information. Patients should be encouraged to frequent forums that are moderated by health care professionals.

The following are suggestions for critical reading of content and participation in online discussions in a community, blog, or forum:

The community website must have clear rules of conduct. Each member must carefully read and follow the rules of the community, blog, or forum. To protect the interests of the community members, administrators must deny access to users with interests different from those of the group (ie, spam, advertising, misinformation, or irrelevant information). The presence of moderators in a community should ensure that all members follow the rules. Channels should exist for reporting inappropriate behavior.

Privacy must be protected. Members of the community, blog, or forum should have the option to remain anonymous or share only the personal information they deem appropriate; it is important to remind patients that many threads and posts are open.

The website must share only validated content. Confirm that the content is supported by bibliographic references to scientific evidence that was produced under expert supervision and that the content is updated regularly.

Comments and posts by individuals can provide useful impetus, but it is important that users realize that the personal experiences of others may not apply to their specific situations. They must be able to confirm the information with authoritative sources and discuss it with their physician. Information about MS treatment discussed online may refer to solutions that have not been approved by the regulatory agencies in a patient’s homeland. Patients and caregivers should be encouraged to consult the website of the national MS organization for official information on drugs approved for MS in their country. In addition, they should ask their physician when questions arise. Moreover, it is important to stress at the outset that the relationship between the patient and the clinician is essential for all aspects of diagnosis and clinical management. Online support groups are not a substitute for direct interaction with the clinician. A survey of 8586 patients with MS revealed that, whereas the first source of information for most patients is the Internet, the vast majority of patients with MS still consider their physician to be the most trusted source for medical information [21]. Given the risks associated with improper treatment of MS, health professionals should take measures to ensure that their patients are prepared and equipped to navigate the eHealth world safely.

## Abbreviations

HON: Health On the Net
MS: multiple sclerosis
SMMS: Social Media and Multiple Sclerosis
